# Treatment costs associated with hospital-based intravenous immunoglobulin therapy compared to home-based subcutaneous immunoglobulin therapy in a cohort of paediatric patients with primary immunodeficiency

**DOI:** 10.1186/1710-1492-7-S2-A25

**Published:** 2011-11-14

**Authors:** Thierry Ducruet, Marie-Claude Levasseur, Anne Des Roches, Renée Dicaire, Elie Haddad

**Affiliations:** 1 Unité de recherche clinique appliquée, CHU Sainte-Justine, Montreal, QC, H3T 1C5, Canada; 2 CHU Sainte-Justine, Department of Pediatrics, University of Montreal, Montreal, QC, H3T 1C5, Canada

## Objectives

To assess the direct and indirect costs associated with hospital-based IVIg therapy compared to home-based SCIg in a cohort of patients with PID from hospital/government and family perspectives.

## Methods

This is a retrospective study on a cohort of patients with PID. Annual costs associated with IVg therapy were assessed for each patient for the year prior to commencing home-based subcutaneous SCIg therapy and the year after for the costs associated with the SCIg therapy. Demographic and clinical information were collected from patient’s chart. Economic data were captured from administrative databases, patients, families, and healthcare providers. Paired t-tests and non-parametric Wilcoxon tests were used to assess the difference in costs.

## Results

Twenty-eight children were enrolled in the study. Mean (SD) age at initial diagnosis was 6.9 (4.2) years. Median duration since Ig therapy initiation was 24 months. The study showed a decrease in costs when shifting from IVIg to SCIg therapy from hospital/government and patients perspectives. The mean difference in costs between the two periods was -$ 4,346 (-$ 6,097; -$ 2,597), p<0.001. The mean difference was statistically significant for both government and family perspectives: -$ 2,602 (p<0.001) and -$ 1,744 (p=-0.002), respectively. The absence of difference in hospital admissions confirms that both strategies are similar in effectiveness and safety.

## Conclusions

The home-based subcutaneous therapy is a safe and cost-effective strategy. Shifting from hospital-based IVIg therapy to home-based SCIg therapy resulted in decreasing costs from both hospital/government and family perspectives. Long-term impact on the health system is substantial.

